# Cardiac Resynchronization Therapy With Defibrillator Using the JROAD-DPC Database: Cost-Effectiveness Analysis

**DOI:** 10.2196/94073

**Published:** 2026-07-21

**Authors:** Kazuki Ohashi, Masaya Watanabe, Yasuhiro Morii, Hisashi Yokoshiki, Kengo Kusano, Katsuhiko Imai, Masahiko Takagi, Teiichi Yamane, Hiroshi Tada, Katsuhiko Ogasawara

**Affiliations:** 1 Faculty of Health Sciences, Hokkaido University, N12-W5, Kita-ku, Sapporo, Hokkaido, 0600812, Japan, 81 011-706-3409; 2 Department of Cardiovascular Medicine, Caress Memorial Hospital, Sapporo, Hokkaido, Japan; 3 Department of Cardiovascular Medicine, Graduate School of Medicine, Hokkaido University, Sapporo, Hokkaido, Japan; 4 Center for Outcomes Research and Economic Evaluation for Health, National Institute of Public Health, Wako, Saitama, Japan; 5 Department of Cardiovascular Medicine, Sapporo City General Hospital, Sapporo, Hokkaido, Japan; 6 Department of Cardiovascular Medicine, National Cerebral and Cardiovascular Center, Suita, Osaka, Japan; 7 Department of Cardiovascular Surgery, Kure Medical Center, Kure, Hiroshima, Japan; 8 Department of Cardiovascular Surgery, Hiroshima University, Hiroshima, Hiroshima, Japan; 9 Division of Cardiac Arrhythmia, Kansai Medical University Medical Centre, Moriguchi, Osaka, Japan; 10 Department of Cardiology, Jikei University School of Medicine, Tokyo Minato-ku, Tokyo, Japan; 11 Department of Cardiovascular Medicine, Faculty of Medical Sciences, University of Fukui, Fukui, Fukui, Japan; 12 Faculty of Engineering, Muroran Institute of Technology, Muroran, Hokkaido, Japan

**Keywords:** cost-effectiveness analysis, cardiac resynchronization therapy with defibrillator, heart failure and reduced ejection fraction, JROAD-DPC database, Japan

## Abstract

**Background:**

Cardiac resynchronization therapy with defibrillator (CRT-D) improves survival, reduces hospitalization, and enhances quality of life in patients with heart failure and reduced ejection fraction (HFrEF). As heart failure prevalence increases in aging societies such as Japan, the associated clinical and economic burden continues to rise. Previous cost-effectiveness analyses conducted in multiple countries indicate that CRT-D may be cost-effective in selected patients with HFrEF. However, its cost-effectiveness within the Japanese health care system remains uncertain.

**Objective:**

This study aimed to evaluate the cost-effectiveness of CRT-D in patients with HFrEF within the Japanese health care setting.

**Methods:**

A partitioned survival model was developed with 3 health states: after treatment (follow-up), hospitalization, and death. Survival for CRT-D was estimated by reconstructing individual patient-level data from the Kaplan-Meier curve of the RAFT (Resynchronization-Defibrillation for Ambulatory Heart Failure Trial) study using the method proposed by Guyot et al followed by fitting multiple parametric models; the gamma distribution was selected for the base case analysis. Survival for optimal medical therapy (OMT), the comparator, was estimated by applying a hazard ratio from a published meta-analysis. Hospitalization rates and device longevity were derived from prior studies. Cost estimates were obtained from the JROAD-DPC (Japanese Registry Of All cardiac and vascular Disease-Diagnostic Procedure Combination) database and the Japanese medical fee schedule. Utility values were assigned according to New York Heart Association class assuming treatment-specific distributions. The analysis was conducted from the public health care payer perspective using a monthly cycle over a 20-year time horizon. Deterministic and probabilistic sensitivity analyses were performed. Additionally, scenario analyses were conducted by varying the duration of treatment effect.

**Results:**

In the base case analysis, per capita costs were ¥12,258,410 (¥1=US $0.006 as of July 7, 2026) for CRT-D and ¥640,056.90 for OMT, resulting in an incremental cost of ¥11,618,353. CRT-D generated 7.07 quality-adjusted life years (QALYs) compared with 4.75 QALYs for OMT, yielding an incremental gain of 2.32 QALYs. The incremental cost-effectiveness ratio (ICER) was ¥5,009,880 per QALY. Scenario analyses showed that, when treatment effect waned after 7.5 years, the ICER increased to ¥5,423,235 per QALY. When the time horizon was shortened to 10 years or extended to 30 years, the ICERs were ¥8,523,072 and ¥4,386,803 per QALY, respectively. Deterministic sensitivity analysis identified CRT-D efficacy (hazard ratio), discount rate, and initial treatment cost as primary ICER drivers. Probabilistic sensitivity analysis produced a median ICER of ¥5,022,618 (IQR ¥4,448,306–¥5,760,425) per QALY, with a 95% credible interval of ¥3,804,418 to ¥7,178,795. At a willingness-to-pay value of ¥5,000,000 per QALY, CRT-D had a 49.2% probability of being cost-effective.

**Conclusions:**

CRT-D demonstrated acceptable cost-effectiveness in patients with HFrEF in Japan. Treatment efficacy and initial cost were the primary determinants of economic value, emphasizing the importance of appropriate patient selection and strategies to reduce device costs.

## Introduction

Cardiac resynchronization therapy with defibrillator (CRT-D) is an established device-based treatment for patients with heart failure (HF) and reduced ejection fraction (HFrEF) [1]. By integrating biventricular pacing with defibrillation capability, CRT-D reduces all-cause mortality, prevents sudden cardiac death, decreases hospitalization for HF, and improves functional status and quality of life in appropriately selected patients [2,3]. Population aging in high-income countries, including Japan, has accelerated the increase in HF prevalence, resulting in a growing population eligible for CRT-D implantation [4,5]. In Japan, earlier reports estimated HF prevalence at approximately 1% [6,7]; however, recent analyses using large administrative claims databases have reported substantially higher estimates of 3% to 6.5% [8]. The European Society of Cardiology estimates that HF accounts for up to 2% of total health care expenditures in high-income countries [9]. These findings indicate a rising HF burden with significant implications for health care spending and resource allocation.

Given constrained health care resources, prioritizing cost-effective therapies is essential. Cost-effectiveness studies of CRT-D conducted outside Japan have produced mixed results, and its economic value remains controversial. Existing evidence indicates that cost-effectiveness varies according to clinical characteristics, including QRS duration, left bundle branch block (LBBB) status, and ischemic vs nonischemic etiology [10-12]. CRT-D has demonstrated cost-effectiveness in patients with QRS duration of 150 ms or higher and New York Heart Association (NYHA) class II to III symptoms, whereas in patients with more advanced HF, such as NYHA class IV, cardiac resynchronization therapy with pacemaker may represent a more economically favorable option [11]. However, most evaluations have been conducted in Western health care systems, limiting generalizability to Japan because of differences in reimbursement structures and cost frameworks. In Japan, cost-effectiveness studies of CRT-D remain limited, and existing health technology assessments have primarily evaluated incremental device features such as automated optimization algorithms and reactive atrial-based antitachycardia pacing (rATP) [13,14]. Therefore, this study aimed to evaluate the cost-effectiveness of CRT-D in the Japanese health care setting using large-scale registry data to provide real-world evidence supporting clinical and policy decision-making.

## Methods

### Overview

The analysis was conducted from the public health care payer perspective using a monthly cycle over a 20-year time horizon, consistent with Japanese guidelines [15]. A reference value of ¥5,000,000 (¥1=US $0.006 as of July 7, 2026) per quality-adjusted life year (QALY) was used to evaluate cost-effectiveness. The incremental cost-effectiveness ratio (ICER) of CRT-D was assessed relative to optimal medical therapy (OMT).

### Study Population

The modeled cohort reflected the CRT-D group of the RAFT (Resynchronization-Defibrillation for Ambulatory Heart Failure Trial) study [16]. The mean age was 66.3 (SD 9.3) years, and 84.8% of the participants were male. NYHA functional class distribution was 76.7% class II and 23.3% class III. The mean left ventricular ejection fraction was 22.5% (SD 5.4%), and mean QRS duration was 158.1 (SD 23.8) ms. Ischemic etiology accounted for 68.7% of cases, and LBBB morphology was present in 71.7%.

### Model Structure and Inputs

A partitioned survival model was developed to evaluate CRT-D cost-effectiveness in patients with HFrEF, with OMT as the comparator. The model included 3 health states: after treatment (follow-up), hospitalization, and death. Hospitalization events included admissions due to cardiac arrest (CA), worsening HF, and CRT-D–related complications. The CRT-D survival curve was derived by digitizing Kaplan-Meier data from long-term RAFT follow-up [16] and reconstructing individual patient-level survival data using the method proposed by Guyot et al [17]. Parametric survival models, including exponential, Weibull, Gompertz, log-logistic, log-normal, gamma, and generalized gamma distributions, were evaluated. On the basis of the Akaike information criterion (AIC), Bayesian information criterion (BIC), and visual inspection, the gamma distribution provided the best fit and was selected. Comparative AIC and BIC results can be found in Table S1 in Multimedia Appendix 1. Survival for OMT was estimated by applying a hazard ratio (HR) for all-cause mortality from the meta-analysis by Woods et al [3] (0.54, 95% CI 0.43-0.69), assuming proportional hazards.

The primary outcome was QALYs, integrating survival and health-related quality of life. Utility values were assigned according to NYHA functional class and differed by treatment group. Adverse outcomes were modeled as hospitalization events, including HF, CA, device-related complications, and temporary disutility associated with device replacement. The incidence of HF hospitalization was obtained from a prior study [18], which reported cumulative rates of 19.5% in the CRT-D group and 26.1% in the OMT group over a median follow-up of 40 months. These cumulative incidences were converted into monthly transition probabilities for each treatment group, assuming a constant hazard throughout the observation period. The incidence of hospitalization due to CA was estimated from indirect published evidence [19-21]. In the CRT-D group, Tseng et al [19] reported that 6.4% of all-cause deaths following cardiac implantable electronic device therapy were attributable to CA. On the basis of the annual all-cause mortality reported in the MADIT-RIT (Multicenter Automatic Defibrillator Implantation Trial–Reduce Inappropriate Therapy) trial, this proportion corresponded to an estimated CA incidence of 0.14% per year in the CRT-D group. In the OMT group, the annual incidence of CA was derived from a previously reported rate of sudden cardiac death in the conventional therapy group (4.5% per year) [21], used as a surrogate marker for arrhythmic CA requiring hospitalization. This yielded an estimated annual incidence of 6.51%. Device-related complications were modeled exclusively in the CRT-D group during follow-up, with a cumulative incidence of 14% over 48 months [22]. This value was converted into a monthly transition probability assuming a constant hazard. Among previously reported complications [23,24], superficial and systemic infections accounted for 21.7% and 26.6%, respectively, whereas lead dislodgement accounted for 51.7%. Device replacement was assumed in cases of systemic infection or lead dislodgement. Device longevity was assumed to be 111 months based on a recent large-scale observational report from a single manufacturer [25]. In the model, device replacements due to battery depletion were assumed to recur continuously without upper limits as long as the simulated cohort survived.

We included costs associated with the initial intervention; device replacement; hospitalizations (CA, HF, and device-related complications); and additional follow-up, including remote monitoring and inappropriate shocks. Costs related to procedure-related complications at initial implantation were incorporated into the initial intervention cost. Cost inputs were derived from analyses of the JROAD-DPC database (approval 20240001) and the Japanese medical fee schedule, which provides standardized reimbursement for each medical service [26]. The database, administered by the Japanese Circulation Society, contains diagnosis procedure combination and per-diem payment system data from approximately 1500 hospitals as of 2024 and covers 2012 to 2024 [27,28]. It represents one of the largest nationwide databases for cardiac and vascular diseases in Japan. For the cost analysis in this study, we extracted data on Japanese patients who underwent initial CRT-D implantation or device replacement between 2012 and 2022. Cost extraction was based on diagnostic and procedure codes. CRT-D implantation and device replacement were identified using procedure codes in the claims database. Hospitalization costs for HF and CA were assumed to be identical between groups as they reflect baseline disease management. Clinical cost differences between groups were captured by adding device-related complications to the CRT-D group. Device-related complications were classified as superficial or systemic infections using *International Classification of Diseases, 10th Revision*, diagnostic codes. The specific codes used for each definition are provided in Table S2 in Multimedia Appendix 1. Utilities were assigned according to NYHA functional class (I-IV), and cohort-level utility was calculated as a weighted average based on the distribution of NYHA classes [29]. Because treatment was assumed to modify the NYHA class distribution, different stable-state utility values were applied to each treatment group. Specifically, CRT-D was assumed to improve NYHA functional class compared with OMT. On the basis of the COMPANION (Comparison of Medical Therapy, Pacing, and Defibrillation in Heart Failure) trial [30], the proportion of patients with NYHA class improvement at 6 months was 57% in the CRT-D group and 38% in the OMT group. These improvement rates were applied to the baseline NYHA class distribution to estimate follow-up utility. The magnitude of improvement was assumed to be equivalent for patients with baseline NYHA class II and III, and the difference in improvement rates between treatment groups was assumed to persist throughout the time horizon. On the basis of these assumptions, the mean health-related utility during follow-up was 0.805 for CRT-D and 0.781 for OMT. For hospitalization events, mean utility values were 0.649 for HF hospitalization and 0.44 for CA hospitalization. Disutility values of −0.10 for complications and –0.05 for device replacement were applied [31]. All model inputs are summarized in Table 1. Costs and utilities were discounted at an annual rate of 2%, consistent with Japanese cost-effectiveness analysis guidelines [15]. All analyses were conducted using R (version 4.4.3; R Foundation for Statistical Computing).

**Table 1.** Summary of model inputs (¥1=US $0.006 as of July 7, 2026).

Parameter and group	Values	Distribution	Source
Hazard ratio (95% CI)^a^	0.54 (0.43 to 0.69)	Log-normal	[3]
Events rate per month (%)		Beta	
HF^b^			
CRT-D^c^	0.54 (0.46 to 0.62)		[19]
OMT^d^	0.75 (0.66 to 0.86)		[19]
CA^e^			
CRT-D	0.01^f^		[19,20]
OMT	0.38 (0.22 to 0.67)		[21]
Complication—CRT-D	0.31 (0.27 to 0.36)		Assumption
Cost (¥)		Gamma	
Initial treatment—CRT-D	6,607,498 (5,469,735 to 7,745,261)		DPC^g^
Device replacement—CRT-D	4,817,601 (3,603,617 to 6,031,585)		DPC
HF—Both	795,777 (636,622 to 954,932)		DPC
CA—Both	719,335 (575,468 to 863,202)		DPC
Complication—CRT-D	6,296,972 (5,037,577 to 7,556,366)		Assumption
Additional follow-up—CRT-D	4900 (–20% to +20%)		Assumption
Utility		Beta	
Follow-up			
CRT-D	0.805 (0.792 to 0.820)		[30]
OMT	0.781		[30]
HF—Both	0.649 (–20% to +20%)		Assumption
CA—Both	0.44 (–20% to +20%)		Assumption
Complication—CRT-D	–0.1		[31]
Replacement—CRT-D	–0.05		[31]
Death—Both	0		—^h^
Others			
Device longevity (mo)			
CRT-D	111 (95 to 126)	Triangular	[25]
Discount rate (%)			
Both	2 (0 to 4)	Beta	[15]

a Unless otherwise specified, the lower and upper values represent the parameter bounds used for the sensitivity analysis and do not represent 95% CIs or 95% credible intervals.

b HF: heart failure.

c CRT-D: cardiac resynchronization therapy with defibrillator.

d OMT: optimal medical therapy.

e CA: cardiac arrest.

f Unless otherwise specified, the lower and upper values represent the parameter bounds used for sensitivity analyses and do not represent 95% CIs or 95% credible intervals. Confidence or credible intervals are not applicable to fixed model parameters.

g DPC: Diagnostic Procedure Combination.

h Not applicable.

### Scenario and Sensitivity Analysis

Formal subgroup analyses were not performed because the individual patient-level clinical characteristics required to define subgroups were unavailable in the data sources. Instead, heterogeneity in treatment effects was evaluated through sensitivity analyses by varying key parameters, including the HR of CRT-D, across plausible ranges reported in the literature.

Scenario analyses examined uncertainty related to treatment effect duration and time horizon. In one scenario, the treatment effect remained constant for 7.5 years and then declined linearly up to 20 years, consistent with prior studies [12,32]. Specifically, this decline was modeled by allowing the HR to approach 1.0 and the difference in utility values between the groups to approach 0. In separate scenarios, the time horizon was set to 10 and 30 years, whereas the treatment effect remained constant and identical to the base case, which used a 20-year horizon.

To evaluate parameter-level and overall model uncertainty, both deterministic sensitivity analysis (DSA) and probabilistic sensitivity analysis (PSA) were performed. Parameter ranges and probability distributions were applied from published sources when available. For parameters lacking reported ranges or distributions, a –20% to +20% variation from base case values was assumed. The discount rate was varied between 0% and 4% in accordance with relevant guidelines [15].

### Ethical Considerations

This study was reviewed and approved by the institutional review board of the Japanese Heart Rhythm Society (approved 20240001) This study used anonymized data from the JROAD-DPC registry. The requirement for individual informed consent was waived by the Institutional Review Board, and participants were provided with the opportunity to opt out in accordance with the applicable ethical guidelines.

## Results

### Base Case and Scenario Analysis

In the base case analysis, per capita costs were ¥12,258,410 for CRT-D and ¥640,056.90 for OMT, yielding an incremental cost of ¥11,618,353. CRT-D produced 7.07 QALYs compared with 4.75 QALYs for OMT, corresponding to an incremental gain of 2.32 QALYs. The resulting ICER was ¥5,009,880 per QALY (Table 2).

**Table 2.** Results of base case and scenario analyses (¥1=US $0.006 as of July 7, 2026).

	Total cost (¥)	Incremental cost (¥)	Total QALYs^a^	Incremental QALYs	ICER^b^ (cost per QALY)
Base case analysis					
CRT-D^c^	12,258,410	11,618,353	7.07	2.32	5,009,880
OMT^d^	640,056.90	―^e^	4.75	―	―
Scenario analysis^f^					
CRT-D	12,258,410	11,597,980	7.04	2.14	5,423,235
OMT	660,429.90	―	4.9	―	―

a QALY: quality-adjusted life year.

b ICER: incremental cost-effectiveness ratio.

c CRT-D: cardiac resynchronization therapy with defibrillator.

d OMT: optimal medical therapy.

e Not applicable.

f The treatment effect was assumed to remain constant for 7.5 years and then decline linearly up to 20 years.

In the scenario analysis, the ICER increased to ¥5,423,235 per QALY when the treatment effect was assumed to decline after 7.5 years (Table 2). When the time horizon was shortened to 10 years, the ICER increased to ¥8,523,072 per QALY. Conversely, extending the time horizon to 30 years reduced the ICER to ¥4,386,803 per QALY.

### Sensitivity Analysis

**Figure 1.**
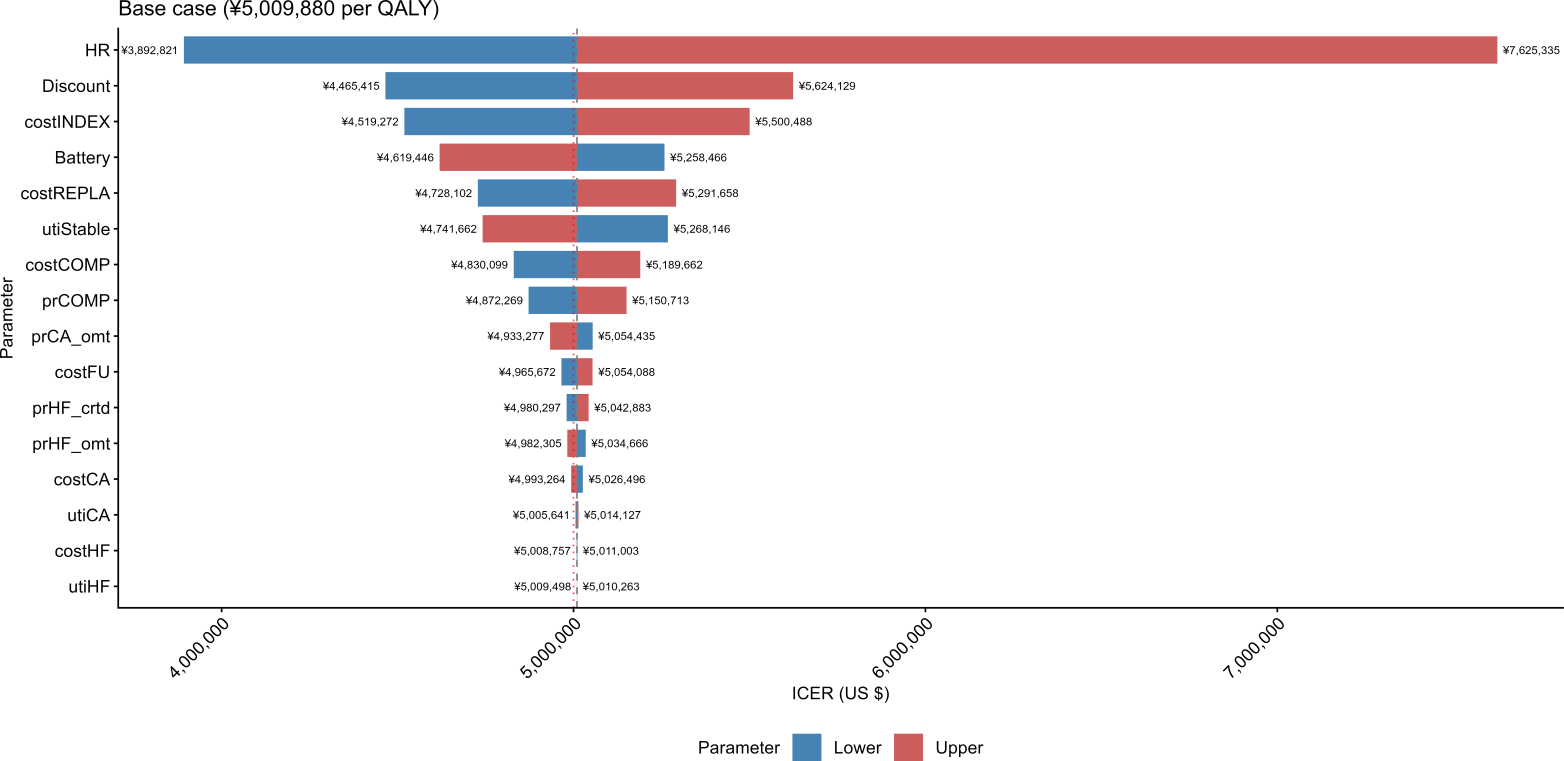
Tornado diagram of the deterministic sensitivity analysis. This diagram depicts the effect of varying individual parameters on the incremental cost-effectiveness ratio (ICER). Bar width represents the ICER range obtained when each parameter varied between its lower and upper bounds. The vertical dotted line denotes the base case ICER of ¥5,009,880 (¥1=US $0.006 as of July 7, 2026) per quality-adjusted life year (QALY). Parameters are ordered from top to bottom according to their relative influence on the ICER. costCA: cost of hospitalization for cardiac arrest; costCOMP: cost of hospitalization for complications; costFU: additional follow-up cost; costHF: cost of hospitalization for heart failure; costINDEX: initial treatment cost; costREPLA: device replacement cost; HR: hazard ratio; prCA_crtd: probability of cardiac arrest in the CRT-D group; prCA_omt: probability of cardiac arrest in the OMT group; prCOMP: probability of hospitalization for complications; prHF_crtd: probability of heart failure in the CRT-D group; prHF_omt: probability of heart failure in the OMT group; utiCA: utility of the cardiac arrest state; utiHF: utility of the heart failure state; utiStable: utility of the stable state.

DSA identified the primary drivers of the ICER as CRT-D efficacy (HR), discount rate, and initial treatment cost (“costINDEX”; Figure 1). Variation in CRT-D efficacy produced ICERs ranging from ¥3,892,821 to ¥7,625,335 per QALY. Variation in the discount rate resulted in ICERs ranging from ¥4,465,415 to ¥5,624,129 per QALY, whereas variation in initial treatment cost yielded ICERs ranging from ¥4,519,272 to ¥5,500,488 per QALY.

**Figure 2.**
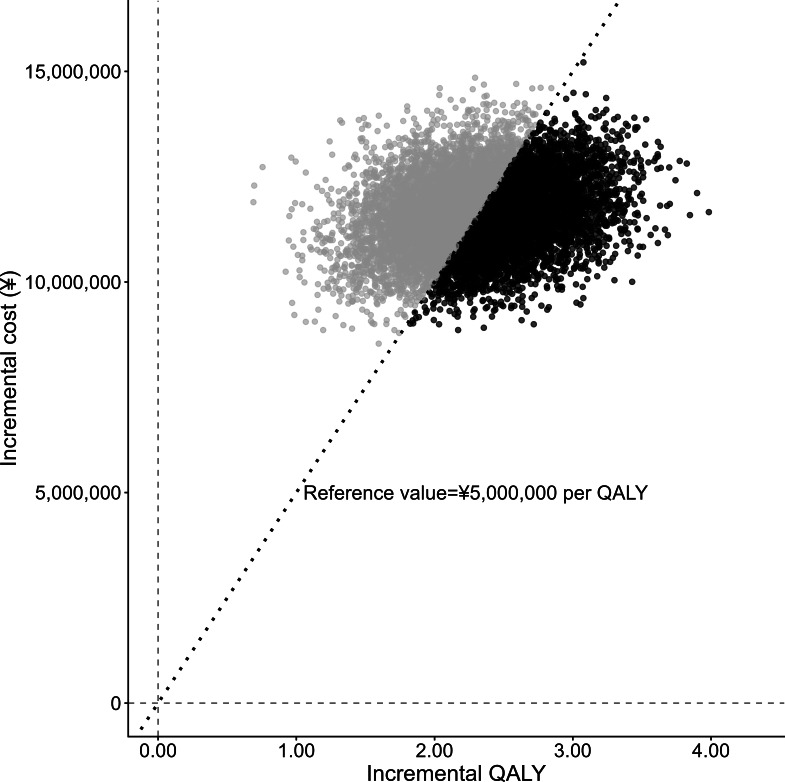
Probabilistic sensitivity analysis scatterplot. This figure presents incremental costs vs incremental quality-adjusted life years (QALYs) across 10,000 simulations. The dotted line indicates the willingness-to-pay threshold of ¥5,000,000 per QALY (¥1=US $0.006 as of July 7, 2026). Black dots represent simulations below the reference value (cost-effective), whereas gray dots indicate simulations exceeding the threshold.

PSA results are shown in Figure 2. The median ICER was ¥5,022,618 per QALY, with a range of ¥3,804,418 to ¥7,178,795 per QALY across the 2.5th and 97.5th percentiles. At a willingness-to-pay threshold of ¥5,000,000 per QALY, the probability that CRT-D was cost-effective was 49.2%.

## Discussion

### Principal Findings

Using the Japanese JROAD-DPC database, we assessed the cost-effectiveness of CRT-D compared with OMT from a public health insurance perspective. Over a 20-year horizon, CRT-D generated an incremental cost of ¥11,618,353 and a QALY gain of 2.32, yielding an ICER of ¥5,009,880 per QALY. This estimate approximated the Japanese reference value. When a waning treatment effect was incorporated, the ICER increased to ¥5,349,656 per QALY, remaining comparable to the reference threshold. In UK settings, Mealing et al [11] reported that CRT-D was cost-effective in only 10 of 24 patient subgroups at a £30,000 per QALY threshold (£1=US $1.34 as of July 7, 2026), highlighting substantial heterogeneity. In 4 subgroups similar to our population (QRS duration of ≥150 ms and NYHA class II and III with or without LBBB), the ICER for CRT-D vs OMT ranged from £15,000 to £20,000, below the accepted UK threshold of £30,000. In a US study [12] using a reference value of US $100,000 per QALY, CRT-D was consistently the most cost-effective strategy among patients with QRS duration above 150 ms regardless of LBBB status or NYHA class. Although reference thresholds differ across the United Kingdom, United States, and Japan, our findings appear conservative in this international context. An ICER of approximately ¥5 million per QALY suggests that further gains in cost-effectiveness will likely depend on refined patient selection.

DSA identified populations in which higher economic value may be achieved. The principal ICER driver was the HR, indicating that greater treatment effects improve cost-effectiveness. A meta-analysis by Woods et al [3] showed that, compared with OMT, the HR for CRT-D was lower in female than in male patients irrespective of QRS duration or age. The same analysis demonstrated that, when CRT-D was compared with OMT, the HR approached 1.0 among patients without LBBB or with QRS duration below 150 ms, indicating attenuation of survival benefit in these subgroups. Because survival was modeled using all-cause mortality, CRT-D is likely to be more cost-effective in patients with a lower burden of high-mortality comorbidities. This interpretation aligns with a recent patient-level meta-analysis of 8 randomized trials [33], which reported substantial survival benefit in patients with no comorbidities (adjusted HR 0.54) but diminished benefit in those with 3 or more comorbidities (adjusted HR 0.83). Accordingly, patients with a lower comorbidity burden may tend to exhibit a more favorable ICER, although this trend requires careful interpretation regarding cumulative longitudinal costs. However, a detailed analysis of specific comorbidities was not feasible in the present study, and further research is warranted to evaluate cost-effectiveness stratified by comorbidity profiles. Additionally, the crucial trials underpinning our efficacy estimates were conducted over a decade ago. Since then, contemporary OMT has advanced significantly with modern foundational therapies (ie, angiotensin receptor–neprilysin inhibitors and sodium-glucose cotransporter 2 [SGLT2] inhibitors) [34,35]. In particular, SGLT2 inhibitors are now widely used in Japan as a key component of guideline-directed medical therapy [36]. If these modern drugs substantially improve baseline outcomes, the incremental benefit of CRT-D over OMT might be attenuated in current practice, potentially increasing the ICER. Future modeling studies incorporating clinical data from the modern therapy era are warranted. Recent incremental innovations in CRT-D, including rATP and optimization algorithms, have improved efficacy but increased device costs. Several studies have shown these enhancements to be cost-effective in Japanese and other national settings [13,14,37]. Overall, the additional costs associated with these functional upgrades appear acceptable from a cost-effectiveness perspective. Another influential determinant was the initial treatment cost, driven largely by device price. The current reimbursement for CRT-D devices in Japan is between ¥3,090,000 and ¥4,750,000 depending on the model. Together with lead costs, this accounts for more than half of the initial treatment cost. Device price also affects downstream costs related to battery depletion and infection requiring replacement; therefore, reductions in device price would likely generate substantial savings. In PSA, the 95% credible interval for the ICER was ¥3,804,418 to ¥7,178,795, with a 49.2% probability of remaining below ¥5 million per QALY. These findings indicate moderate parameter uncertainty, with ICER estimates distributed within approximately –¥2.5 million to +¥2.5 million of the reference value.

### Limitations

Several limitations of this study should be acknowledged. First, although the JROAD-DPC database captures a substantial proportion of acute care hospitals in Japan, it does not include all medical institutions. Furthermore, the JROAD-DPC database was not used to derive clinical event probabilities because this database possesses inherent limitations in tracking long-term, longitudinal patient outcomes across different medical institutions once patients are discharged to outpatient care. Consequently, cost inputs may be subject to selection bias, limiting generalizability to all patients with HF in Japan. Second, the 20-year time horizon required extrapolation of survival and costs beyond observed follow-up. To mitigate this limitation, survival curves were generated using parametric modeling of long-term data from the RAFT study. Although using overseas trial data (RAFT) requires caution regarding generalizability, the trial’s mean age (66.3 years) is comparable to that of major Japanese CRT-D registries (66.5 or 68.4 years) [38]. Nevertheless, this population-level cost-effectiveness model does not incorporate individual risk factors. Future Japanese studies incorporating patient-level subgroup analyses are needed to refine estimates and support value-based allocation within the health care system. Finally, detailed subgroup analyses by clinical characteristics, such as QRS duration, LBBB status, or comorbidity burden, were not feasible because of data constraints. Instead, the potential influence of these factors was examined through DSA by varying HRs rather than directly estimating subgroup-specific effects.

Despite these limitations, to our knowledge, this study represents one of the first comprehensive cost-effectiveness evaluations of CRT-D in the Japanese health care setting using real-world cost data.

### Conclusions

CRT-D for patients with HFrEF in Japan demonstrated cost-effectiveness within an acceptable range relative to the reference value. Sensitivity analyses identified the HR and initial treatment cost as key determinants of value. These findings emphasize the importance of identifying patient subgroups with a high expected treatment response. Specifically, future cost-effectiveness analyses stratified by comorbidity profiles will provide further valuable insights. From a policy perspective, reducing device costs may further enhance the societal value of this intervention.

## Supplementary material

10.2196/94073 Multimedia Appendix 1 Goodness-of-fit statistics (Akaike information criterion and Bayesian information criterion) for candidate parametric survival models used for survival extrapolation, observed Kaplan-Meier curve and fitted parametric survival models, and coding definitions used to identify interventions and complications in the claims database.

## Abbreviations

AIC: Akaike information criterion
BIC: Bayesian information criterion
CA: cardiac arrest
COMPANION: Comparison of Medical Therapy, Pacing, and Defibrillation in Heart Failure
CRT-D: cardiac resynchronization therapy with defibrillator
DSA: deterministic sensitivity analysis
HF: heart failure
HFrEF: heart failure and reduced ejection fraction
HR: hazard ratio
ICER: incremental cost-effectiveness ratio
JROAD-DPC: Japanese Registry Of All cardiac and vascular Disease-Diagnostic Procedure Combination
LBBB: left bundle branch block
MADIT-RIT: Multicenter Automatic Defibrillator Implantation Trial–Reduce Inappropriate Therapy
NYHA: New York Heart Association
OMT: optimal medical therapy
PSA: probabilistic sensitivity analysis
QALY: quality-adjusted life year
RAFT: Resynchronization-Defibrillation for Ambulatory Heart Failure Trial
rATP: reactive atrial-based antitachycardia pacing
SGLT2: sodium-glucose cotransporter 2
