# Supplementary material for: Cardiac Resynchronization Therapy With Defibrillator Using the JROAD-DPC Database: Cost-Effectiveness Analysis
Source: Interact J Med Res. 2026 Jul 21;15:e94073. doi: 10.2196/94073 (PMC13387419; doi:10.2196/94073)
Supplement: Multimedia Appendix 1 [file ijmr-v15-e94073-s001.docx]

| Models | AIC | BIC |
| --- | --- | --- |
| Exponential | 2522.1 | 2526.3 |
| Weibull | 2496.7 | 2505.2 |
| Gompertz | 2500.6 | 2509.1 |
| Log-logistic | 2510.5 | 2519.0 |
| Log-normal | 2556.5 | 2526.3 |
| Gamma | 2499.4 | 2507.9 |
| Generalize gamma | 2498.0 | 2510.7 |

Table S1. Survival Model Fitting Results

AIC, Akaike information criteria; BIC, Bayesian information criteria

Figure S1. Comparison of Fitted Parametric Survival Models


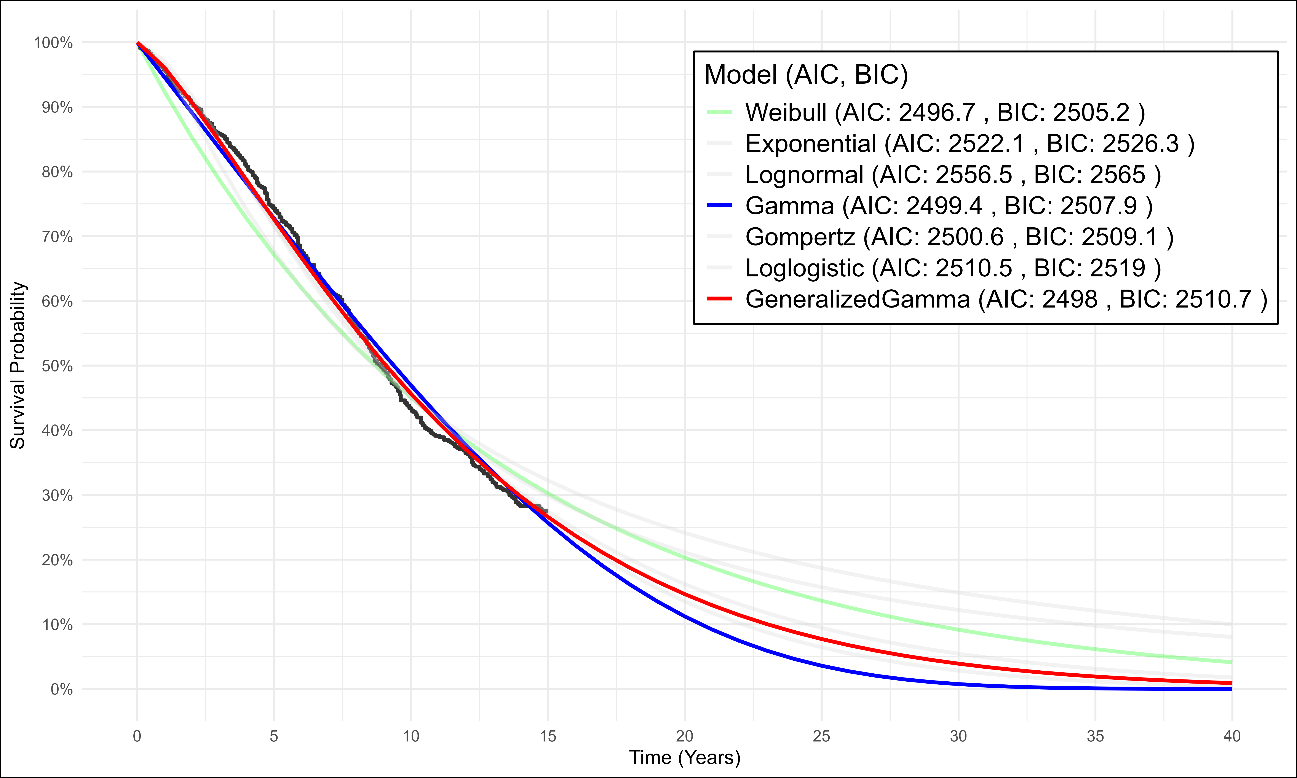


Table S2

| Items | Definition |
| --- | --- |
| Initial intervention | SPC: K599-3 |
| Device replacement | SPC: K599-4 |
| Complication |  |
| Infection | ICD-10: T814, T941, T793, T827-3 |
| Sepsis | ICD-10: A41.0, A41.1, A41.9 |
| Infectious endocaritis | ICD-10: I400, SCD: GL3P |

ICD-10, International Classification of Diseases-10; SCD, Standardized Disease Code; SPC, surgical procedure code
